# Tdap vaccination during pregnancy and risk of chorioamnionitis and related infant outcomes

**DOI:** 10.1016/j.vaccine.2023.04.043

**Published:** 2023-04-26

**Authors:** Victoria Greenberg, Gabriela Vazquez-Benitez, Elyse O. Kharbanda, Matthew F. Daley, Hung Fu Tseng, Nicola P. Klein, Allison L. Naleway, Joshua T.B. Williams, James Donahue, Lisa Jackson, Eric Weintraub, Heather Lipkind, Malini B. DeSilva

**Affiliations:** a MedStar Washington Hospital Center, Washington, DC, United States; b HealthPartners Institute, Bloomington, MN, United States; c Institute for Health Research, Kaiser Permanente Colorado, Denver, CO, United States; d Kaiser Permanente Southern California, Pasadena, CA, United States; e Kaiser Permanente Vaccine Study Center, Oakland, CA, United States; f Center for Health Research, Kaiser Permanente Northwest, Portland, OR, United States; g Ambulatory Care Services, Denver Health, Denver, CO, United States; h Marshfield Clinic, Research Institute, Marshfield, WI, United States; i Kaiser Permanente Washington, Seattle, WA, United States; j Immunization Safety Office, U.S. Centers for Disease Control and Prevention, Atlanta, GA, United States; k Weill Cornell Medicine, New York, NY, United States; l HealthPartners Institute, Bloomington, MN, United States

**Keywords:** Vaccination, Chorioamnionitis, Pregnancy, Infant

## Abstract

**Introduction:**

An increased risk of chorioamnionitis in people receiving tetanus toxoid, reduced diphtheria toxoid, and acellular pertussis (Tdap) vaccine during pregnancy has been reported. The importance of this association is unclear as additional study has not demonstrated increased adverse infant outcomes associated with Tdap vaccination in pregnancy.

**Methods:**

We conducted a retrospective observational cohort study of pregnant people ages 15–49 years with singleton pregnancies ending in live birth who were members of 8 Vaccine Safety Datalink (VSD) sites during October 2016–September 2018. We used a time-dependent covariate Cox model with stabilized inverse probability weights applied to evaluate associations between Tdap vaccination during pregnancy and chorioamnionitis and preterm birth outcomes. We used Poisson regression with robust variance with stabilized inverse probability weights applied to evaluate the association of Tdap vaccination with adverse infant outcomes. We performed medical record reviews on a random sample of patients with *ICD-10-CM*-diagnosed chorioamnionitis to determine positive predictive values (PPV) of coded chorioamnionitisfor “probable clinical chorioamnionitis,” “possible clinical chorioamnionitis,” or “histologic chorioamnionitis.”

**Results:**

We included 118,211 pregnant people; 103,258 (87%) received Tdap vaccine during pregnancy; 8098 (7%) were diagnosed with chorioamnionitis. The adjusted hazard ratio for chorioamnionitis in the Tdap vaccine-exposed group compared to unexposed was 0.96 (95% CI 0.90–1.03). There was no association between Tdap vaccine and preterm birth or adverse infant outcomes associated with chorioamnionitis. Chart reviews were performed for 528 pregnant people with chorioamnionitis. The PPV for clinical (probable or possible clinical chorioamnionitis) was 48% and 59% for histologic chorioamnionitis. The PPV for the combined outcome of clinical or histologic chorioamnionitis was 81%.

**Conclusions and relevance:**

Tdap vaccine exposure during pregnancy was not associated with chorioamnionitis, preterm birth, or adverse infant outcomes. *ICD-10* codes for chorioamnionitis lack specificity for clinical chorioamnionitis and should be a recognized limitation when interpreting results.

## 1. Introduction

Pertussis, also known as whooping cough, is a highly contagious infection caused by the *Bordatella pertussis* bacterium. Newborns are at the highest risk for morbidity and mortality due to immune system immaturity. Pertussis can be prevented by tetanus toxoid, reduced diphtheria toxoid, and acellular pertussis (Tdap) vaccine administration. Vaccination during pregnancy results in transplacental passage of protective antibodies and is recommended in each pregnancy by the U.S. Advisory Committee on Immunization Practices (ACIP) and the American College of Obstetricians and Gynecologists (ACOG) [1,2,3]. Tdap vaccination has been found to be highly effective in preventing neonatal pertussis [4]. Overall, Tdap vaccination during pregnancy has been deemed safe [5]. However, a small but statistically significant increased risk of chorioamnionitis diagnosis in pregnant people vaccinated with the Tdap vaccine during pregnancy has previously been reported, although this finding is not consistent across studies [6–8]. Further investigation of the association of Tdap vaccination in pregnancy and chorioamnionitis is important.

Chorioamnionitis is an intrauterine infection of maternal-fetal origin or fetal origin alone [9,10]. Neonates born to pregnant people with chorioamnionitis are at higher risk of preterm birth [3,11] and subsequent morbidity, such as neonatal sepsis [12–15] and pneumonia [14], as well as to longer-term sequelae, including bronchopulmonary dysplasia [16] and cerebral palsy [17]. Despite the observed associations between Tdap vaccination during pregnancy and chorioamnionitis, the impact of this association is unclear as additional work has not demonstrated increased risk for adverse infant outcomes associated with Tdap vaccination during pregnancy [7,8,18]. Multiple factors may contribute to the observed association of chorioamnionitis with receipt of Tdap vaccine during pregnancy such as outcome misclassification or immortal time bias. We sought to further evaluate the association between receipt of Tdap vaccine during pregnancy with chorioamnionitis, preterm birth, and related adverse infant outcomes and to determine the validity of diagnostically coded chorioamnionitis.

## 2. Material and methods

### 2.1. Study design

We conducted a retrospective, observational cohort study of pregnant people ages 15–49 years with singleton pregnancies ending in live birth identified within the VSD during October 1, 2016–September 30, 2018 and a pregnancy start date during April 14, 2016 to December 24, 2017. The primary aim was to evaluate the association between Tdap vaccine administration during pregnancy and chorioamnionitis, preterm birth, and adverse infant outcomes associated with chorioamnionitis. A secondary aim was to determine the validity of *International Classification of Diseases, Tenth Revision Clinical Modification (ICD-10)-CM* codes for chorioamnionitis using chart review.

### 2.2. Setting and study population

The VSD is a collaborative project between the CDC’s Immunization Safety Office and several integrated health systems representing approximately 3% of the U.S. population [19]. The study population included pregnant people aged 15–49 years with singleton pregnancies ending in a live birth and enrollment in 1 of 8 VSD integrated healthcare organizations (Kaiser Permanente: Washington, Northwest, Northern California, Southern California, and Colorado; HealthPartners; Marshfield Clinic; and Denver Health). This study was approved by institutional review boards of all participating healthcare organization sites with a waiver of informed consent and was conducted consistent with federal law and CDC policy. §§ See e.g., 45 C.F.R. part 46.102(l)(2), 21 C.F.R. part 56; 42 U.S.C. §241(d); 5 U.S.C. §552a; 44 U.S.C. §3501 et seq.

Pregnancies were identified using a validated algorithm based on administrative, electronic health record (EHR), and claims data [20]. We included pregnant people with singleton live births and a pregnancy start date during April 14, 2016 to December 24, 2017 who were continuously insured from 6 months prior to their last menstrual period through 6 weeks postpartum and had at least one outpatient visit in a VSD health care system during pregnancy. For analysis of infant outcomes infants were required to have enrollment starting at birth hospitalization and followed through the first month of life. Pregnant persons and infants were linked through site-specific algorithms.

### 2.3. Exposure

Tdap vaccination information was captured at VSD sites through EHR, claims data, and bidirectional linkages with state and local immunization registries [21].

### 2.4. Outcomes

Chorioamnionitis identified by *ICD-10-CM* codes (i.e., ICD-10: O41.12, O41.12x, O41.121x, O41.122x, O41.123x, O41.129x) assigned during delivery hospitalization was the primary outcome. We identified infant outcomes of transient tachypnea of newborn, neonatal sepsis, pneumonia, respiratory distress syndrome, and convulsions in the newborn using diagnosis codes in the first month of life, while preterm birth (<37 weeks) was based on birth records (Supplementary Table 1). These adverse infant outcomes are known to be associated with chorioamnionitis.

Trained abstractors performed medical record reviews on a random sample of patients with ICD-10-CM diagnosis of chorioamnionitis stratified by site, Tdap vaccine administration, and presence of preterm delivery defined as <37 weeks’ gestation. Three pediatric and maternal-fetal medicine physicians (VG, HSL, MD) then adjudicated presumptive cases to determine whether they met definitions for “probable clinical chorioamnionitis” or “possible clinical chorioamnionitis,” adapted from the Global Alliance of Immunization safety Assessment in pregnancy (GAIA) clinical case definition for chorioamnionitis [22]. We designated cases meeting definitions for either “probable” or “possible” clinical chorioamnionitis as confirmed cases. We defined “probable clinical chorioamnionitis” as maternal temperature ≥ 38 °C (100.4°F) on one occasion during delivery hospitalization plus one or more of the following: 1) Baseline fetal tachycardia (FHR > 160 bpm for 10 min or longer, excluding accelerations, decelerations and periods of marked variability or, where continuous monitoring is not available, an FHR exceeding 160 bpm during and after at least three consecutive contractions), 2) maternal white blood cell count (WBC) ≥ 15,000 per mm^3^ in the absence of corticosteroids, or 3) definite purulent fluid from the cervical os. We defined “possible clinical chorioamnionitis” as maternal temperature 38 °C on one occasion during delivery hospitalization plus either documented maternal tachycardia (HR > 100 bpm) or uterine tenderness and does not otherwise meet criteria for probable chorioamnionitis. We also evaluated whether cases met the case definition of “histologic chorioamnionitis” based on findings from pathological review of the placenta.

### 2.5. Additional variables

Covariates included age, VSD site, race-ethnicity, percent of households below the 150% federal poverty level in the Census tract [23], receipt of influenza vaccine during pregnancy, adequacy of prenatal care utilization defined using the Kotelchuck Index [24], and comorbidities of pregnant people (e.g., smoking, pregestational diabetes mellitus, chronic hypertension, obesity, systemic lupus erythematous, coagulation defects, alcohol/drug dependence, smoking, and listeria infection) associated with increased risk for chorioamnionitis (Supplementary Table 2), identified using ICD-10-CM diagnoses.

### 2.6. Statistical analyses

We described the frequencies of baseline categorical variables between Tdap vaccine-exposed and unexposed pregnant people and used standardized mean differences to evaluate whether distributions were similar between groups. Standardized mean differences above 0.20 were considered potential confounders. We constructed a propensity score for Tdap vaccine receipt including the following covariates: age at last menstrual period (LMP), gravidity, smoking status, percent of households below the 150% federal poverty level, race, ethnicity, influenza vaccination during pregnancy, Kotelchuck Index, and VSD site as main effects and calculated the stabilized inverse probability weights (SIPW). We further evaluated whether covariates were balanced after applying the SIPW.

We evaluated the associations between Tdap vaccine administered at any time during pregnancy and chorioamnionitis, preterm birth, and adverse infant outcomes. For chorioamnionitis and preterm birth, we used a time-dependent covariate Cox regression model with gestational age in days as the time scale. For the adverse infant outcomes evaluated, we used Poisson regression with robust variance with the general estimating equation method. SIPW were applied in both regression models.

For the chart review, we calculated PPVs and 95% confidence intervals for “probable clinical chorioamnionitis,” the combined outcome of “probable or possible clinical chorioamnionitis,” “histologic chorioamnionitis,” and all case definitions combined overall and stratified by preterm delivery. All analyses were performed using SAS ([SAS/STAT] software, Version [9.4], Cary, NC: SAS Institute Inc.

## 3. Results

**Fig. 1.**
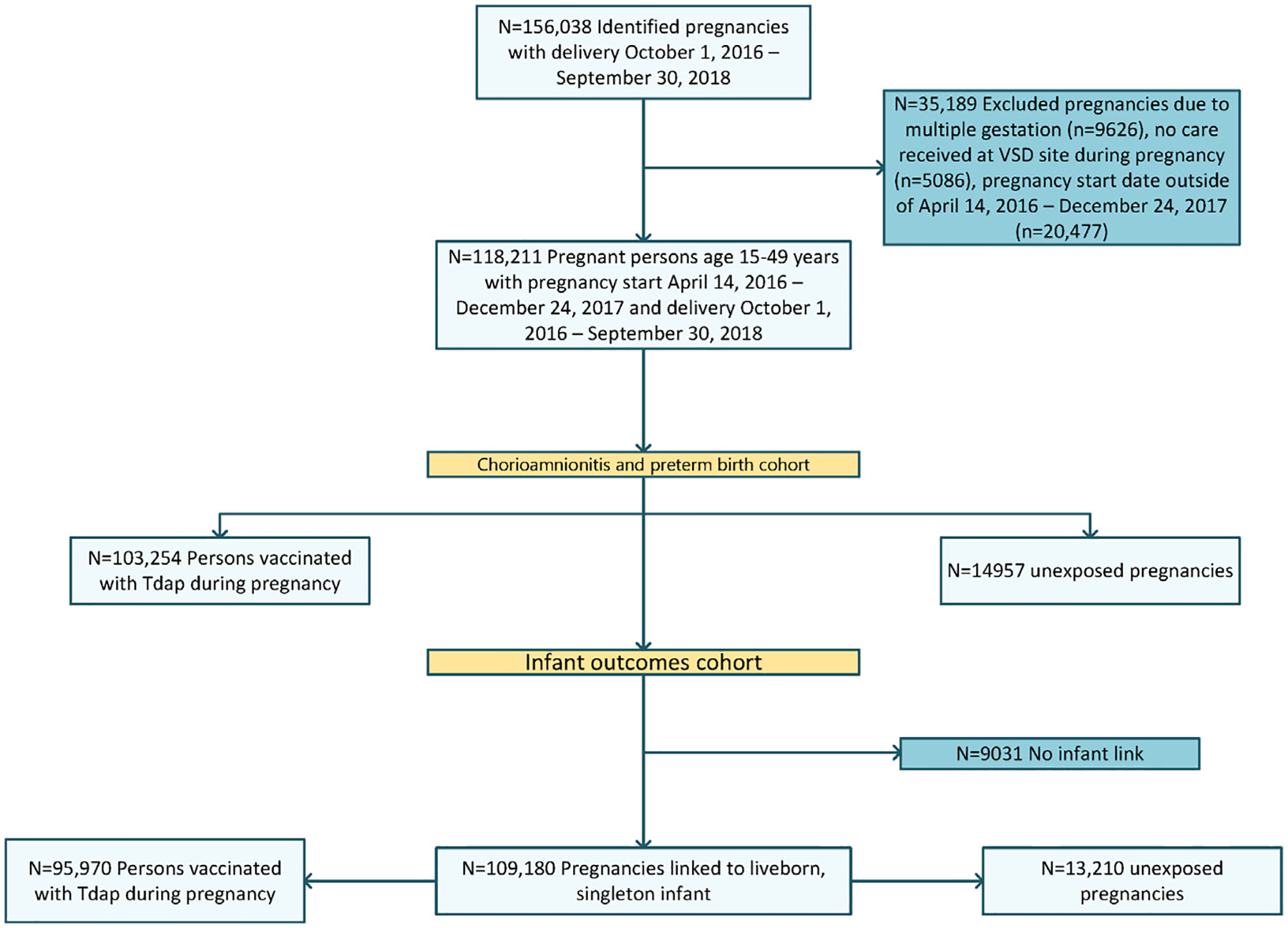
Flow chart of pregnancies ending in delivery of singleton, liveborn infant during October 1, 2016 – September 30, 2018 identified at 8 Vaccine Safety Datalink sites with separate cohorts for evaluation of association between Tdap vaccine administration during pregnancy and 1) chorioamnionitis and preterm birth and 2) selected infant outcomes.

We identified 118,211 pregnant people with live births from October 1, 2016–September 30, 2018 and pregnancy start dates of April 4, 2016 to December 24, 2017 for the analysis of chorioamnionitis and preterm birth, and 109,180 pregnancies linked to a liveborn, singleton infant for the infant outcomes (Fig. 1). Among the entire study population, 103,254 (87%) received Tdap vaccine during pregnancy (Table 1). Of these Tdap vaccine administrations during pregnancy, 87% were Adacel, 11% were Boostrix, and 2% were unknown vaccine manufacturer. Receipt of Tdap vaccine occurred at 27 weeks’ gestation or later in 98% of those vaccinated. The majority of pregnant people were between 25 and 34 years of age. The highest percentage of both Tdap vaccinated and unvaccinated pregnant people were non-Hispanic White followed by Hispanic. Tdap unvaccinated people had higher rates of inadequate prenatal care and lower rates of influenza vaccine receipt during pregnancy. We did not observe any covariate imbalances after applying propensity weights (Supplemental Fig. 1).

Chorioamnionitis was diagnosed in 8098 (7%) of pregnancies, among whom 427 (5%) delivered <37 weeks. The adjusted hazard ratio (HR) for chorioamnionitis after receipt of Tdap vaccine was 0.96 (95% CI 0.90–1.03) (Table 2). Supplemental Figure 2 shows the incidence of chorioamnionitis by week following Tdap vaccine administration during pregnancy or if unvaccinated. There was no observed clustering of chorioamnionitis at a specific interval following Tdap vaccine administration during pregnancy. In the unvaccinated group, there was a bimodal distribution of chorioamnionitis with increased incidence in those delivering prematurely (peak around 28 weeks) and those with post-term delivery (i.e., after 40 weeks’ gestation). A higher percentage of adverse infant outcomes were identified in the Tdap vaccine-unexposed group compared to the Tdap vaccine-exposed group (Table 2). In adjusted analyses, there was no association between Tdap vaccine adminis tration during pregnancy and preterm birth, adjusted HR 1.01 (95% CI: 0.96–1.07) or adverse infant outcomes.

Chart reviews were performed for 528 pregnant people with *ICD-10-CM* diagnosed chorioamnionitis. The PPV for clinical chorioamnionitis was less than 50% for the entire chart review sample (Table 3). Thirty-one percent met the case definition for probable clinical chorioamnionitis (n = 169) and 48% met the case definition for either probable or possible clinical chorioamnionitis (n = 258). The diagnosis of probable or possible clinical chorioamnionitis was confirmed for a higher percentage of term births (54%, n = 223) compared to preterm births (28%, n = 35). The PPV for histologic chorioamnionitis was 59% (n = 315) overall and higher in preterm births (72%, n = 91). Chorioamnionitis was only a histologic diagnosis in 52% (n = 280) of cases. Among all *ICD-10-CM* diagnosed chorioamnionitis cases, 69% were sent for pathologic review and results were available for all but 8 cases. The placenta was sent for pathology review in 83% of preterm deliveries compared to 65% of term deliveries. For all births, the combined outcome of clinical (possible or probable) or histologic chorioamnionitis had a PPV of 81%.

## 4. Discussion

In this evaluation of over 100,000 pregnancies, Tdap vaccine exposure during pregnancy was not associated with chorioamnionitis, preterm birth, or adverse infant outcomes. The lack of association between Tdap vaccine receipt during pregnancy and chorioamnionitis is consistent with other VSD studies that did not find an increased risk of adverse infant outcomes following antenatal Tdap vaccine exposure [7]. Over the last decade, Tdap vaccine administration during pregnancy has increased [25], with over 80% of pregnant people in our cohort receiving the vaccine as recommended, at or after 27 weeks’ gestation. The incidence of pertussis in infants below 6 months of age decreased by 42% after Tdap vaccination became routinely recommended during pregnancy in 2013 [4]. Administration of Tdap vaccine during pregnancy is an important public health strategy to reduce severe pertussis infections in infants.

The previously observed associations of chorioamnionitis with receipt of Tdap vaccine during pregnancy did not account for immortal time bias [6–8]. Thus, our previous results [7] should be recontextualized in light of our new findings related to the importance of accounting for immortal time bias in the analytic approach. During pregnancy, immortal time bias may be present when an exposure (e.g., receipt of Tdap vaccine) depends on the duration of the pregnancy. Pregnant people who deliver before the recommended time period for Tdap vaccine (i.e., 27–36 weeks’ gestation) will be classified as unexposed because they did not remain pregnant long enough to have the exposure even if they intended to receive the vaccine later in pregnancy [26]. People who give birth at later gestational ages typically have more prenatal visits and thus more opportunities to receive the Tdap vaccine during pregnancy. Additionally, we identified a bimodal distribution of chorioamnionitis diagnosis based on gestational age in the unvaccinated group. One previous study found that chorioamnionitis complicates nearly one third of patients with preterm labor. [27] Ninety-eight percent of our cohort received Tdap vaccination at 27 weeks’ gestation or later. Thus, both the outcome, chorioamnionitis, and exposure, Tdap vaccination, are time dependent during pregnancy making it important to account for immortal time bias. [6–8] Adjusting for immortal time bias in this analysis allowed us to reduce time interval-based error in estimating the association between the exposure (Tdap vaccine) and outcome (chorioamnionitis). Other studies evaluating drug or vaccine exposures during pregnancy in which outcomes studied vary by gestational age have used a similar analytic approach [26,28–30].

We also found that the ICD-10 codes for chorioamnionitis lack specificity for clinical chorioamnionitis based on chart review results. The definition of chorioamnionitis is inconsistent between clinicians and across epidemiologic studies [31]. In a previous study by the Vaccine Safety Datalink (VSD) evaluating the association of Tdap vaccination during pregnancy and chorioamnionitis, the positive predictive value (PPV) of the ICD-9 code 658.41 for “possible clinical chorioamnionitis,” defined as maternal temperature ≥ 38.0 °C and one additional clinical finding, was 0.78 (95% confidence interval [CI], 0.72, 0.83) [6]. In this evaluation, the PPV for clinical chorioamnionitis was even lower at 48%, resulting in outcome misclassification.

Although we were unable to differentiate between clinical and histologic chorioamnionitis in our primary analysis, the chart abstractions we performed allowed us to distinguish cases which only met criteria for histologic chorioamnionitis. Histologic chorioamnionitis, which does not reliably predict adverse infant outcomes [32–34], has the same diagnostic code as clinical chorioamnionitis and can be reported in the absence of clinical signs and symptoms of infection or positive cultures from the placenta, membranes, or amniotic fluid. In these cases, the inflammatory changes in the membranes may result from noninfectious insults, such as hypoxic injury, trauma, meconium, or allergens [22]. Additionally, ICD-10 diagnostic codes for chorioamnionitis may be added to the patient chart after placental pathologic results are available, further reducing the specificity of diagnostic coding for identifying clinical chorioamnionitis [35]. The clinical significance of histologic chorioamnionitis alone is uncertain, which may explain the lack of associated adverse infant outcomes. Future studies should conduct quantitative bias analyses to estimate the impact of outcome misclassification [36].

Several important limitations should be noted. This study cohort was limited to pregnant people with continuous insurance coverage who had at least one outpatient prenatal visit, and complete pregnancy, delivery, and neonatal health data available. This may limit generalizability, particularly to higher-risk individuals with intermittent or no medical insurance and lack of prenatal care. Although we adjusted for outcome misclassification and immortal time bias in the analysis, there may have been residual confounding related to differences in healthcare-seeking behavior between vaccinated and unvaccinated individuals [37]. Additionally, there are regional differences in peripartum care, chorioamnionitis diagnosis, and treatment. Third, by focusing only on live births, we may introduce selection bias which is problematic as chorioamnionitis diagnoses are also associated with non-live birth outcomes. Additional bias can occur because documentation and diagnostic coding often captures more severe forms of disease, including comorbidities assessed, creating potential for residual confounding [38]. We could not ensure that complete clinical data was available in the electronic health record (e.g., report of purulent fluid from cervical os or uterine tenderness), which may decrease the number of individuals meeting criteria for clinical chorioamnionitis.

## 5. Conclusion

Receipt of Tdap vaccine during pregnancy was not associated with chorioamnionitis, preterm birth, or adverse infant outcomes in a large cohort of pregnant people with singleton pregnancies ending in live birth from an administrative dataset. Our study provides additional reassuring data regarding the safety of Tdap vaccine administration during pregnancy. Tdap vaccination during pregnancy should continue to be encouraged given the vaccine’s effectiveness in preventing infant pertussis and reassuring safety profile. Future studies evaluating chorioamnionitis as an outcome should include adjustments for immortal time bias and acknowledge the heterogeneity of diagnostic coding for chorioamnionitis when interpreting results.

## Supplementary Material

Appendix A. Supplementary data

## Figures and Tables

**Table 1:** Baseline characteristics of Tdap vaccine exposed and unexposed pregnant people with a live singleton birth at 8 Vaccine Safety Datalink sites, October 1, 2016–September 20, 2018.

	Mother-infant cohort, N = 118,211	
	Tdap vaccine-unexposed^a^ (n = 14,957; 12.7%)	Tdap vaccine-exposed** (n = 103,254; 87.3%)
* **Age at delivery, years**		
<18	72 (0.5)	467 (0.5)
18–24	2,253 (15.1)	12,432 (12.0)
25–34	8,605 (57.5)	61,880 (59.9)
≥35	4,027 (26.9)	28,475 (27.6)
* **Race/Ethnicity**		
Asian	1,234 (8.3)	17,940 (17.4)
Black	1,545 (10.3)	5,774 (5.6)
Hispanic	5406 (36.1)	36,178 (35.0)
Other	927 (6.2)	5,355 (5.2)
White	5,845 (39.1)	38,007 (36.8)
* **Year of delivery**		
2016	89 (0.6)	155 (0.2)
2017	8,106 (54.2)	57,984 (56.2)
2018	6,762 (45.2)	45,116 (43.7)
* **Prenatal Care Index**		
Adequate/plus	9,754 (65.2)	73,495 (71.2)
Intermediate	3,754 (25.1)	27,109 (26.3)
Inadequate	1,449 (9.7)	2,650 (2.6)
**Received influenza vaccine during pregnancy**	3,627 (24.3)	68,912 (66.7)
* **Medical co-morbidities**		
Smoking	2,727 (18.3)	15,875 (15.4)
Pre-gestational diabetes mellitus	309 (2.1)	2,148 (2.1)
Chronic hypertension	926 (6.2)	5955 (5.8)
BMI ≥ 30	38,633 (25.8)	26,106 (25.3)
Gestational diabetes mellitus	1,628 (10.9)	12,131 (11.8)
Hypertensive disorders of pregnancy***	2,406 (16.1)	17,079 (16.5)
* **Vaccine Safety Datalink Site**		
A	4,726 (31.6)	41,565 (40.3)
B	463 (3.1)	4,123 (4.0)
C	625 (4.2)	4,031 (3.9)
D	424 (2.8)	1,263 (1.2)
E	598 (4.0)	4,674 (4.5)
F	288 (1.9)	2,025 (2.0)
G	7,386 (49.4)	43,059 (41.7)
H	447 (3.0)	2,514 (2.4)
**Mean Poverty** (SD)^d^	20.9 (14.8)	19.0 (14.1)

* Indicates variable included in propensity score;

a Tdap vaccine-unexposed may have received Tdap vaccine prior to pregnancy or after delivery;

b Race and ethnicity were classified based on information available in each participating site’s medical record and included in the study due to differences in immunization rates by race and ethnicity;

c diagnoses during inpatient admissions from 6 months before pregnancy through the end of the pregnancy;

d percentage of families in census tract whose income was below 150% of the federal poverty level; SD standard deviation.

** 98% of Tdap vaccine administered at 27 weeks gestational age or later.

*** Includes gestational hypertension and preeclampsia.

**Table 2:** Prevalence of chorioamnionitis, preterm birth, and selected infant outcomes according to receipt of Tdap vaccine and adjusted Hazard ratios (aHR) or adjusted relative risks (RR) with 95% confidence intervals (CI).

Outcome	Tdap vaccine-unexposed n (%)	Tdap vaccine-exposed, n (%)	aHR/aRR (95%CI)
**Chorioamnionitis** ^1^	854 (5.7%)	7244 (7.0%)	0.96 (0.90–1.03)
**Preterm birth (<37 weeks’ gestation)** ^1^	1885 (12.6%)	6087 (5.9%)	1.01 (0.96–1.07)
**Infant outcomes** ^2^			
**Apgar < 7**	226 (2.0%)	790 (0.9%)	1.07 (0.90–1.28)
**Transient tachypnea of newborn**	376 (3.3%)	2426 (2.8%)	1.01 (0.91–1.12)
**Neonatal sepsis**	140 (1.2%)	386 (0.4%)	0.95 (0.75–1.21)
**Pneumonia**	51 (0.4%)	136 (0.2%)	0.67 (0.42–1.07)
**Respiratory distress syndrome**	696 (6.1%)	1648 (1.9%)	1.02 (0.92–1.12)
**Convulsions in newborn**	27 (0.2%)	117 (0.1%)	1.29 (0.84–1.98)

1 Hazard ratios (HR) were estimated using a time-dependent covariate Cox regression;

2 Relative Risks (RR) were estimated using a Poisson regression with robust variance with the general estimating equation method; stabilized inverse probability weights were applied in both regression models.

**Table 3:** Chart review findings for 538 pregnant people with *ICD-10-CM* diagnosed chorioamnionitis.

	All, N = 538		Term (≥37 weeks GA), N = 412		Preterm (<37 weeks GA), N = 126	
	N	PPV	N	PPV	N	PPV
**Probable Clinical Chorioamnionitis** *	169	31%	150	36%	19	15%
Possible** **OR Probable Clinical Chorioamnionitis**	258	48%	223	54%	35	28%
**Histologic chorioamnionitis** ***	315	59%	224	54%	91	72%
**Placenta sent for pathology**	372	69%	268	65%	104	83%
**Pathology results available**	364	68%	262	64%	102	81%
**Possible or probable clinical OR histologic chorioamnionitis**	434	81%	330	80%	104	83%

PPV = Positive predictive value; GA = gestational age.

* Probable clinical chorioamnionitis defined as maternal temperature ≥38 °C (100.4°F) during delivery hospitalization plus one or more of the following: 1) Baseline fetal tachycardia, 2) maternal WBC ≥ 15,000 per mm3 in the absence of corticosteroids, 3) definite purulent fluid from the cervical os.

** Possible clinical chorioamnionitis defined as maternal temperature ≥38 °C (100.4°F) during delivery hospitalization plus either maternal tachycardia (HR > 100 bpm) oruterine tenderness.

*** Histologic chorioamnionitis confirmed by findings on pathologic examination of placenta.

## Data Availability

Data will be made available on request.
