# Supplementary material for: Tdap vaccination during pregnancy and risk of chorioamnionitis and related infant outcomes
Source: Vaccine. Author manuscript; Available in PMC 2023 Aug 30. (PMC10466272; doi:10.1016/j.vaccine.2023.04.043)
Supplement: Appendix A. Supplementary data [file NIHMS1927091-supplement-Appendix_A__Supplementary_data.docx]

**Supplementary Table 1: Pregnancy and infant outcomes evaluated with ICD-9 and ICD-10 codes listed.**

| **Pregnancy outcome** | **ICD-10** | **ICD-9*** |
| --- | --- | --- |
| Chorioamnionitis | O41.12, O41.12x, O41.121x, O41.122x, O41.123x, O41.129x | 658.4, 658.4x |
| Preterm birth <37 weeks’ gestation | N/A – based on birth records | N/A – based on birth records |
| **Infant outcomes** |  |  |
| Transient tachypnea of newborn | P22.1 | 770.6 |
| Neonatal sepsis | P36, P36.x. P36.xx, A40, A40.x, A41, A41.x | 771.8, 771.81, 771.89, 995.91, 995.92 |
| Pneumonia | P23, P23.x, J12-J18 770.0, 480.0-486 | 770.0, 480.0-486 |
| Respiratory distress syndrome | P22.0, P28.5, P28.81 | 769, 770.84, 770.87 |
| Convulsions in newborn | P90 | 779.0 |

*ICD-9 codes listed for historical reference and for countries still using ICD-9 coding.

**Supplementary Table 2. Co-morbid conditions or exposures with increased risk for chorioamnionitis and associated ICD-9 and ICD-10 codes.**

| **Comorbidities** | **ICD-10** | **ICD-9*** |
| --- | --- | --- |
| Diabetes  Pre-existing diabetes in pregnancy  Gestational diabetes | E10.x, E10.xx, E11.x, E11.xx  O24.0, O24.0x, O24.0xx, O24.1, O24.1x, O24.1xx, O24.2, O24.2x, O24.2xx, O24.3, O24.3x, O24.3xx  O24.4xx | 250.xx  251.x,255.xx,  648.0x  648.8x |
| Obesity  Obesity in pregnancy | E66.x  O99.21, O99.21x | 278.xx, V85  649.xx |
| Systemic lupus erythematosus | M32.x, M32.xx | 710.0x, 714, 446 |
| Coagulation defects (includes antiphospholipid ab) | D68.5, D68.5x, D68.6, D68.6x | 649.3x  289.81,289.82 |
| Renal disease | N00.1 – N00.7, N01.x, N02.1 – N02.7, N03.1-N03.7, N04.1-N04.7 | 274.2, 592,593,753  580.xx-588 |
| Hypertension  Gestational hypertension | I11, I12, I13, I15, I16  O10, O11, I10  O12-O16 | 642.0-642.3401-405  642.4-642.7 |
| **Exposures** |  |  |
| Alcohol use/drug dependence | F10-F16.xx; O99.31, O99.31x, O99.32, O99.32x | 303,305, 648.3,655.5 |
| Smoking | F17.x, F17.xx, Z72.0, Z87.891,  O99.33, O99.33x | V15.82,305.1,649.0 |
| **infections** |  |  |
| Listeria | A32, A32.x, A32.xx | 027 |

*ICD-9 codes listed for historical reference and for countries still using ICD-9 coding.

**Figure 1**. Flow chart of pregnancies ending in delivery of singleton, liveborn infant during October 1, 2016 – September 30, 2018 identified at 8 Vaccine Safety Datalink sites with separate cohorts for evaluation of association between Tdap vaccine administration during pregnancy and 1) chorioamnionitis and preterm birth and 2) selected infant outcomes.

**Supplemental Figure 1.** Covariate balance measured by standard mean difference before and after applying propensity weights (WT).


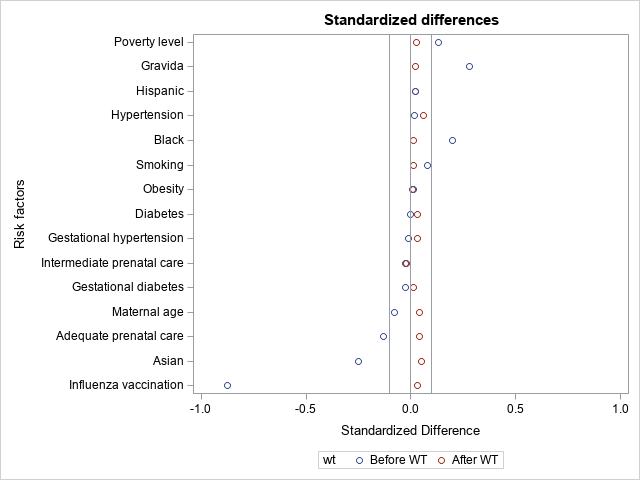


**Supplemental Figure 2.** Incidence of chorioamnionitis by week following Tdap vaccine administration during pregnancy (27–31 weeks, 32–36 weeks, or ≥37 weeks) or if unvaccinated with Tdap vaccine during pregnancy. Gestational age for unvaccinated starts at 27 weeks.
